# Rural-Urban Disparities in Cervical Cancer Incidence and Mortality Among US Women

**DOI:** 10.1001/jamanetworkopen.2024.62634

**Published:** 2025-03-03

**Authors:** Trisha L. Amboree, Haluk Damgacioglu, Elizabeth Y. Chiao, Kathleen M. Schmeler, Kalyani Sonawane, Ashish A. Deshmukh, Jane R. Montealegre

**Affiliations:** 1 Department of Public Health Sciences, Medical University of South Carolina, Charleston; 2 Hollings Cancer Center, Medical University of South Carolina, Charleston; 3 Department of Behavioral Science, The University of Texas MD Anderson Cancer Center, Houston; 4 Department of Epidemiology, The University of Texas MD Anderson Cancer Center, Houston; 5 Department of Medical Oncology, The University of Texas MD Anderson Cancer Center, Houston; 6 Department of Gynecologic Oncology and Reproductive Medicine, The University of Texas MD Anderson Cancer Center, Houston

## Abstract

This cross-sectional study examines rural-urban disparities in cervical cancer incidence and mortality among US women.

## Introduction

Women living in rural areas, particularly minoritized populations, face health care shortages contributing to suboptimal cervical cancer screening and care. Recent evidence shows an increase in cervical cancer incidence in the US^1,2^; however, rural-urban disparities in cervical cancer incidence and mortality remain unclear.

## Methods

In this cross-sectional study, we used the National Program of Cancer Registries and Surveillance, Epidemiology, and End Results (NPCR-SEER) database^3^ to identify cervical cancer cases. Cervical cancer mortality data were obtained from death certificates recorded by the National Center for Health Statistics. Using SEER*Stat version 8.4.3, we estimated annual incidence and 5-year mortality rates per 100 000 women, age-adjusted according to the 2000 US population. Rurality was assessed using the 2013 Rural-Urban Continuum Codes. We corrected rates to account for survey-weighted smoothed hysterectomy prevalence using data from the Behavioral Risk Factor Surveillance System^4^ (eAppendix in Supplement 1). The Joinpoint Regression program version 5.1.0.0 was used to calculate piecewise log-linear trends in incidence and derive annual percentage change (APC) and 95% CIs. This study followed the STROBE reporting guidelines for cross-sectional studies and was deemed exempt by the institutional review board at The Medical University of South Carolina because the data are deidentified and publicly available, in accordance with 45 CFR §46.

## Results

**Figure 1.**
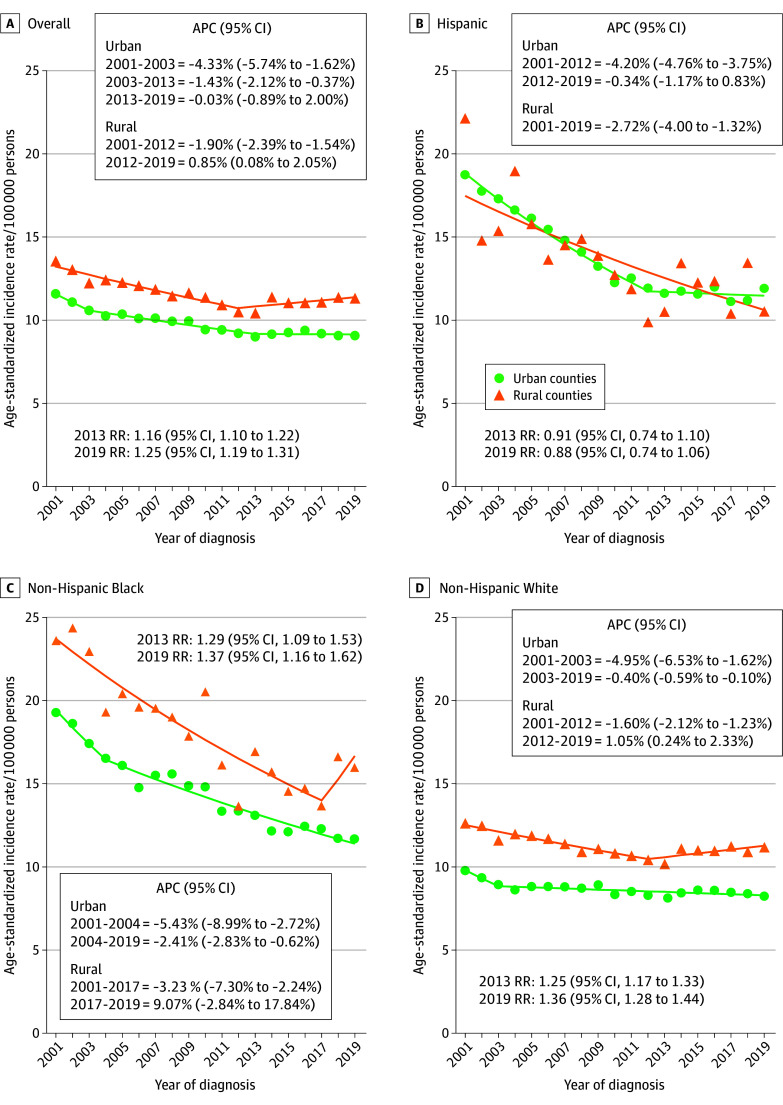
Trends in Hysterectomy-Corrected Cervical Cancer Incidence by Rurality and Race and Ethnicity, 2001-2019 Cervical cancer cases were identified using *International Classification of Diseases Oncology–3 *codes C53.0-C53.9 and histology codes 8010 to 8671 and 8940 to 8941. Rurality was assessed using 2013 Rural-Urban Continuum Codes and categorized as urban (1-3) and rural (4-9). Race and ethnicity were categorized as Hispanic, non-Hispanic Black, and non-Hispanic White, and other. The other category includes (non-Hispanic) American Indian or Alaska Native, Asian or Pacific Islander, and other unspecified; however, data on other race and ethnicity were not presented due to insufficient sample size when disaggregated. Race and ethnicity information was abstracted from medical records. Registries use standardized protocol for both race and ethnicity information, initially collected by health care facilities and practitioners. Rate ratios (RRs) represent rural vs urban incidence rates for each time period, with 95% CIs assuming large-sample normal approximation. The 95% CIs for annual percentage change (APC) were estimated using Joinpoint’s empirical quantile method. The calendar segments reported for each APC were defined according to the identification of calendar years when a change in the APC occurred (ie, Joinpoint models), where the first year represents the earliest year of the segment and the second year represents the latest year of the segment.

During 2001 to 2019, 222 425 cervical cancer cases were identified, of which 84.3% were from urban counties and 59.9% were in non-Hispanic White (hereafter, White) women. Hysterectomy-corrected incidence rates were 11.9 and 10.0 per 100 000 in rural and urban counties, respectively. Overall, incidence increased by 0.85% per year (95% CI, 0.08% to 2.05%) in rural counties from 2012 to 2019 after decreasing from 2001 to 2012. Conversely, incidence plateaued in urban counties (APC for 2013-2019, −0.03%; 95% CI, −0.89% to 2.00%) after decreasing during 2001 to 2013 (Figure 1A). The gap between rural and urban incidence rates widened from 2013 (rate ratio [RR], 1.16; 95% CI, 1.10 to 1.22) to 2019 (RR, 1.25; 95% CI, 1.19 to 1.31). Among rural White women, incidence increased by 1.05% per year (95% CI, 0.24% to 2.33%) during 2012 to 2019 (Figure 1D). APC among rural non-Hispanic Black (hereafter, Black) women was 9.07% per year (95% CI, −2.84% to 17.84%) during 2017 to 2019, but was not statistically significant (Figure 1C). Incidence declined among rural Hispanic women (Figure 1B). Incidence also declined among urban White and Black women and plateaued among urban Hispanic women.

**Figure 2.**
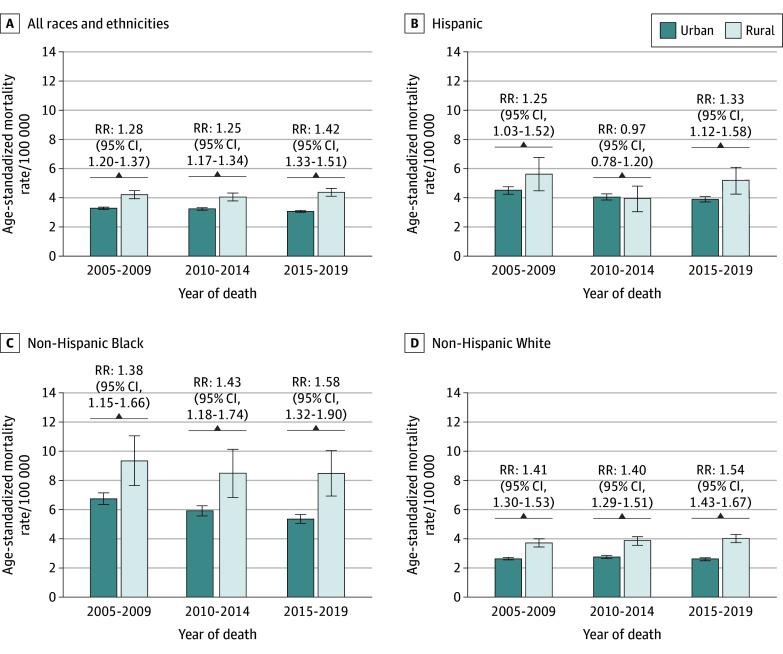
Hysterectomy-Corrected Cervical Cancer Mortality Rates by Rurality and Race and Ethnicity, 2005-2019 Mortality data included only deaths from cervical cancer specifically based on death certificate data ascertained by the National Center for Health Statistics. Rurality was assessed using 2013 Rural-Urban Continuum Codes and categorized as urban (1-3) and rural (4-9). Race and ethnicity were categorized as Hispanic, non-Hispanic Black, and non-Hispanic White, and other. The other category includes (non-Hispanic) American Indian or Alaska Native, Asian or Pacific Islander, and other unspecified; however, data on other race and ethnicity were not presented due to insufficient sample size when disaggregated. Race and ethnicity information was abstracted from medical records. Registries use standardized protocol for both race and ethnicity information, initially collected by health care facilities and practitioners. Error bars indicate 95% CIs for rates using corrected SEs and assuming large-sample normal approximation. Rate ratios (RRs) represent rural vs urban mortality rates for each time period, with 95% CIs using corrected SEs and assuming large-sample normal approximation.

Overall, mortality was 1.42 (95% CI, 1.33 to 1.51) times higher in rural vs urban counties during 2015 to 2019 (Figure 2). Similarly, mortality was higher among rural Hispanic women (RR, 1.33; 95% CI, 1.12 to 1.58), rural Black women (RR, 1.58; 95% CI, 1.32 to 1.90), and rural White women (RR, 1.54; 95% CI, 1.43 to 1.67), compared with their urban counterparts.

## Discussion

This cross-sectional study found a recent increase in cervical cancer incidence in rural US counties, specifically among White women. In addition, incidence was 25% higher and mortality was 42% higher in rural vs urban counties in recent years. The increase in incidence and mortality in rural US counties may reflect lower screening coverage^5^ and lower utilization of diagnostic and therapeutic care, likely resulting from heightened access barriers experienced in rural areas. Additionally, if unaddressed, lower human papillomavirus (HPV) vaccine uptake in rural areas^6^ may contribute to further widening disparities in the future.

A limitation of this study is that county-level data do not account for within-county variation. Furthermore, small sample sizes do not allow for annual mortality trend analyses when stratified by race, ethnicity, and rurality; thus, we estimated 5-year mortality rates and RRs.

The findings from this study highlight the importance of enhancing preventive care (HPV vaccination and screening access) in rural US counties, which remains suboptimal in comparison with their urban counterparts.^5,6^ If differences in preventive care are not urgently mitigated, disparities are likely to magnify in future years.

## Notes

Supplement 1. **eAppendix.** Estimation of hysterectomy prevalence
eReference

Supplement 2.
Data Sharing Statement
