# Supplementary material for: Rural-Urban Disparities in Cervical Cancer Incidence and Mortality Among US Women
Source: JAMA Netw Open. 2025 Mar 3;8(3):e2462634. doi: 10.1001/jamanetworkopen.2024.62634 (PMC11877160; doi:10.1001/jamanetworkopen.2024.62634)
Supplement: Supplement 1. — eAppendix. Estimation of hysterectomy prevalence eReference [file jamanetwopen-e2462634-s001.pdf]

Supplemental Online Content

Amboree TL, Damgacioglu H, Chiao EY, et al. Rural-urban disparities in cervical cancer incidence and mortality among US women. *JAMA Netw Open*. 2025;8(3):e2462634. doi:10.1001/jamanetworkopen.2024.62634

eAppendix. Estimation of hysterectomy prevalence

eReference

This supplemental material has been provided by the authors to give readers additional information about their work.

© 2025 Amboree TL et al. *JAMA Network Open*.

eAppendix.

Estimation of hysterectomy prevalence:

The prevalence of hysterectomy was estimated using biannual (years 2000, 2002, 2004, 2006, 2008, 2010, 2012, 2014, 2016, 2018, 2020) data from the Behavioral Risk Factor Surveillance System (BRFSS). [1] Specifically, the overall and race/ethnicity-specific survey-weighted prevalence of hysterectomy was estimated for even-numbered years by rurality (rural, urban) and 5-year age group (0-4, 5-9, 10-14, 15-19, 20-24, 25-29, 30-34, 35-39, 40-44, 45-49, 50-54, 55-59, 60-64, 65-69, 70-74, 75-79, 80-84, 85+) using the BRFSS survey item: "*Have you had a hysterectomy? A hysterectomy is an operation to remove the uterus (womb).*". Race and ethnicity were categorized as Hispanic, non-Hispanic Black, and non-Hispanic White, and 'Other'. The 'Other' category includes (non-Hispanic) American Indian or Alaska Native, Asian or Pacific Islander, and other unspecified, however data on 'Other' race/ethnicity were not presented due to insufficient sample size when disaggregated. Prevalence for odd-numbered years (2001, 2003, 2005, 2007, 2009, 2011, 2013, 2015, 2017, 2019) was calculated by averaging the estimated prevalence of the flanking even-numbered years. Hysterectomy prevalence was plotted by 5-year age group and rurality, and each plot was fit using a linear regression trendline to obtain a trendline equation (e.g., y= mx+ b, where y= prevalence of hysterectomy, m= slope of the trendline, x= corresponding year, and b= y-intercept of the trendline). All estimates were smoothed to the equation of the trendline.

Additional methods

Rate ratios and 95% CIs were calculated using large-sample normal approximation to quantify geographic disparities in incidence and mortality by race and ethnicity. Statistical significance was defined as $\alpha = .05$.

eReference

1. Centers for Disease Control and Prevention. Behavioral Risk Factor Surveillance System: Annual Survey Data. Accessed April 1, 2024, from https://www.cdc.gov/brfss/annual_data/annual_data.htm

© 2025 Amboree TL et al. *JAMA Network Open*.
