# Supplementary material for: Rural-Urban Disparities in Cervical Cancer Incidence and Mortality Among US Women
Source: JAMA Netw Open. 2025 Mar 3;8(3):e2462634. doi: 10.1001/jamanetworkopen.2024.62634 (PMC11877160; doi:10.1001/jamanetworkopen.2024.62634)
Supplement: Supplement 2. — Data Sharing Statement [file jamanetwopen-e2462634-s002.pdf]

Data Sharing Statement

Amboree. Rural-Urban Disparities in Cervical Cancer Incidence and Mortality Among US Women. *JAMA Netw Open*. Published March 03, 2025.
doi:10.1001/jamanetworkopen.2024.62634

Data
Data available: No

Additional Information
Explanation for why data not available: Data for the National Program of Cancer Registries (NPCR), Surveillance, Epidemiology, and End Results (SEER) database, and Behavioral Risk Factor Surveillance System (BRFSS) are publicly available at www.cdc.gov/cancer/uscs/public-use, https://seer.cancer.gov/data/, and https://www.cdc.gov/brfss/annual_data/annual_data.htm, respectively.
